# COVID 19 vaccination as a trigger of acute genital ulcers in an immunocompromised adolescent—case study and literature review

**DOI:** 10.1186/s12905-024-02930-6

**Published:** 2024-03-05

**Authors:** Karolina Pokora, Karolina Kowalczyk, Robert Peterek, Marlena Cwynar, Rafał Stojko, Paweł Madej, Agnieszka Drosdzol-Cop

**Affiliations:** 1 Department of Endocrinological Gynecology, Medical University of Silesia, Katowice, Poland; 2 Chair and Department of Gynecology, Obstetrics and Gynecological Oncology, School of Health Sciences in Katowice, Medical University of Silesia, Katowice, Poland

**Keywords:** Acute genital ulcers, Lipschütz ulcers, Periodic fever, Aphthous stomatitis, Pharyngitis, And adenitis syndrome, Behcet’s disease, Coronavirus disease of 2019 vaccine, Adolescents

## Abstract

Acute genital ulcers can affect females of all ages. In children, they often appear as an emergency and remain a diagnostic challenge for pediatricians, gynecologists and dermatologists. Prompt diagnosis and identification of disease- related factors help to implement appropriate treatment. Firstly, it is crucial to properly compile the past medical history of the patient. Past infectious, autoimmune, malignant or traumatic conditions, as well as vaccinations may contribute to the occurrence of acute genital ulcers. Moreover, new infectious agents, such as severe acute respiratory syndrome coronavirus 2 and vaccinations against Coronavirus disease of 2019, may play a significant role in the development of atypical clinical symptoms. Here we present a case of a 12-year-old girl with acute genital ulcers. Additional symptoms accompanying the ulcer included: abdominal pain, nausea, vomiting, dysuria, vulvar pain and fever. Blood test showed leukocytosis, especially neutrophilia and monocytosis and increased levels of c-reactive protein and procalcitonin. Serological tests for the most common infections were negative. Moreover, the patient had a history of autoimmune diseases. She had periodic fever, aphthous stomatitis, pharyngitis, and adenitis syndrome, and IgA vasculitis, also known as Henoch-Schönlein purpura in her past medical history. Additionally, she was vaccinated against SARS-CoV-2 shortly before the lesions appeared.

## Background

The most common etiology of suddenly appearing vulvar ulcers in women is sexually transmitted disease. However, these types of lesions may also occur in sexually inactive girls or adolescents [1]. Acute genital ulcers (AGU), also known as Lipschütz acute vulvar ulceration, refer to a non-sexually related condition where a few ulcers suddenly appear in the genital region. The infection mainly triggers conditions which tend to resolve spontaneously [1]. Clinicians from different medical specialties, such as pediatricians, gynecologists, and dermatologists, find it challenging to diagnose adolescent patients.

The lesions are usually located within the labia minora, less frequently in the labia majora, vestibule or perineum [2]. They are well demarcated aphthae of several centimeters which are found in the mucosa and have fibrinous, purulent or necrotic center [2, 3]. Additionally, flu-like symptoms might occur, such as fatigue, fever, and lymphadenitis [4]. AGUs usually resolve within a few weeks. So far, this rare ulcerative condition has not been fully analyzed, but it seems to be of viral etiology [5]. Studies have shown that lesions within the vulva are often concomitant with a systemic infectious disease, where flu-like conditions and infectious mononucleosis are noted most often [1].

**Fig. 1:**
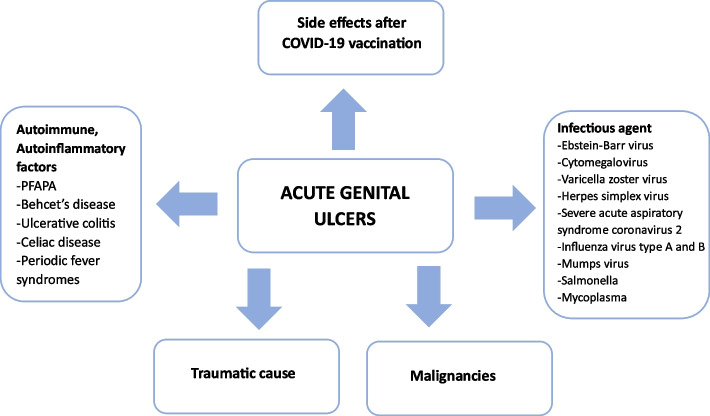
Acute genital ulcers differential diagnosis scheme [9]. PFAPA—periodic fever, aphthous stomatitis, pharyngitis and cervical adenitis

Diagnosis of AGU is based on exclusion of other diseases manifesting with genital ulceration [6, 7]. which are listed in Fig. 1 The detection of pathogenic microorganisms’ genetic material, serologic and antigen tests usually facilitate the diagnosis. In addition, genital ulceration might be a distressing sign of malignancy. However, given the average age of the patients with AGU, neoplastic causes are less likely. Vulvar cancer is considered a disease of older adults, diagnosed at a median age of 68 [8].

Some autoimmune diseases are associated with the risk of ulcers in the pediatric population. These diseases include in particular periodic fever, aphthous stomatitis, pharyngitis, and adenitis (PFAPA) syndrome and Behcet’s disease (BD), which have a similar pathomechanism. Clinical signs of PFAPA syndrome such as pharyngitis, cervical adenitis, aphthous stomatitis, and recurrent fever are used to identify this complex auto-inflammatory condition. Alternating periods of relapses and remissions characterize the disease’s course, and gastrointestinal issues frequently accompany the primary symptoms. After triggering factors occur, ulcers may also develop because of the immunological and genetic variables that created the PFAPA syndrome. Behcet's disease is identified by eye and skin lesions, vaginal and oral aphthosis, and neurological and vascular symptoms.

Additionally, the most common factors causing ulcers include viral diseases, especially infectious mononucleosis syndrome (IMS). It is frequently linked to two viruses, mainly Epstein-Barr virus (EBV) and, less commonly, cytomegalovirus (CMV). Patients with vulvar ulcers and a positive EBV test result show systemic symptoms of IMS, and enlargement of lymph nodes in remote ulcers is also characteristic. In patients with AGU caused by CMV infection, CMV inclusions were observed in vulvar and cervical cells [3].

The present review of literature focuses on a possible causal link between the appearance of AGU and a variety of plausible causes, including infectious, autoimmunological, inflammatory and neoplastic conditions, as well as those following COVID-19 vaccination and more we added other causes of genital ulcers.

## Materials and methods

We collected the main clinicopathological data (age, symptoms, past medical history, circumstances of lesion development, diagnostic test results, treatment management and a follow-up status) of the case of AGU in adolescent patient in our institution (Brothers Hospitallers Hospital in Katowice). The patient and her parents gave a permission to the publication of the article. Furthermore, we conducted a narrative literature review on PubMed database to identify and discuss AGU cases in adolescents, with special focus on autoimmune disorders and vaccinations. The following key words were established: AGU, Lipschütz ulcers, adolescents, COVID-19 vaccine, infection, autoimmune, inflammatory, neoplastic, PFAPA syndrome, Behcet’s disease (BD). We collected 73 scientific articles from 2004 to 2023. We rejected 39 due to different patient's age and discrepancies in clinical symptoms.

### Case report

A 12-year-old patient was admitted to the pediatric gynecology department with burning, itching and pain in the genital area. The girl complained of abdominal pain, nausea, vomiting and dysuria for a few days, later on accompanied by vulvar pain and fever. Her external genitalia were tender, her vulva was inflamed and swollen with bleeding subcutaneous ulcers on the labia minora. Her laboratory test results, indicative of inflammation (e.g. elevated C-reactive protein level (CRP), leukocytosis and neutrophilia) are shown in Table 1. The lesions affected the patient’s labia majora, labia minora, hymenal caruncle and vestibulum. They were separated from the surrounding tissues with a demarcation line (Fig. 1A). Nevertheless, the girl was in a generally good condition. The other mucous membranes were clean and properly moist. Abdominal examination showed no abnormalities. Pediatrician prescribed cefuroxime and analgesics, however, after three days due to symptoms exacerbation she was referred to Pediatric and Adolescents Gynecology Department. The therapy was complemented with topical medication, namely ointment with silver and sulfathiazole, and non-steroidal anti-inflammatory drugs. An ulcerated tissue sample was taken for histopathological examination, which revealed necrotic and purulent lesions with no neoplastic process. The patient was discharged three days later, after the pain had subsided and the wound healing had begun. She continued the antibiotic regimen and topical treatment at home.

**Table 1:** Results of the blood test with serological examination and urinalysis. The underlined results are abnormal

Abnormalities in blood test	Urinalysis	Serological tests
Leucocytes 23.38 × 10^3^/ul [N: 3.98–10.04 × 10^3^/ul]	Color cloudy, dark yellow [N: clear to pale yellow]	HSV IgG antibodies 3.38 (result positive above 1.1)
Hemoglobin 14.4 g/dl [N: 11.8–16 g/dl]	pH 9.0 [N:5–7]	HSV IgM antibodies 0.85 (doubtful result IgM antibodies 0.8–1.1)
Neutrophiles 19.33 × 10^3^/ul [N: 1.82–7,47 × 10^3^/ul]	Gravity 1.005 [N:1.005–1.030]	VZV Negative
Monocytes 2.15 × 10^3^/ul [N: 0.19–0.72 × 10^3^/ul]	Protein 258 mg/dl [N: absent]	Toxoplasmosis Negative
Lymphocytes 1.83 × 10^3^/ul [N:1.16–3.33 × 10^3^/ul]	Sediment Flat epithelia numerous, round epithelia 0-1hpf^a^ [N:1–5 epithelial cells hpf^a^] WBC^b^ 30–40 hpf^a^ [N: 0–5 hpf^a^]	CMV Negative
Eosinophils Absent [N:0.02–0.32 × 10^3^/ul]	Mucus Abundant	HIV Negative
C-reactive protein 59.7 mg/l [N:0-5 mg/l]	Bacteria Abundant	Rubella Negative
Procalcitonin 1.87 ng/ml [N: 0.05–0.1 ng/ml]		SARS-CoV-2 Negative

The underlined results are abnormal. The complete blood count and biochemical values standardized by the laboratory which carried out the mentioned tests and adjusted them to gender and age have been attached

^a^*hpf* per high power field ^b^, *WBC* white blood cells, *CMV* cytomegalovirus, *HIV* human immunodeficiency virus, *HSV* herpes simplex virus, *SARS-CoV-2* severe acute respiratory syndrome coronavirus 2, *VZV* Varicella Zoster Virus

**Fig. 2:**
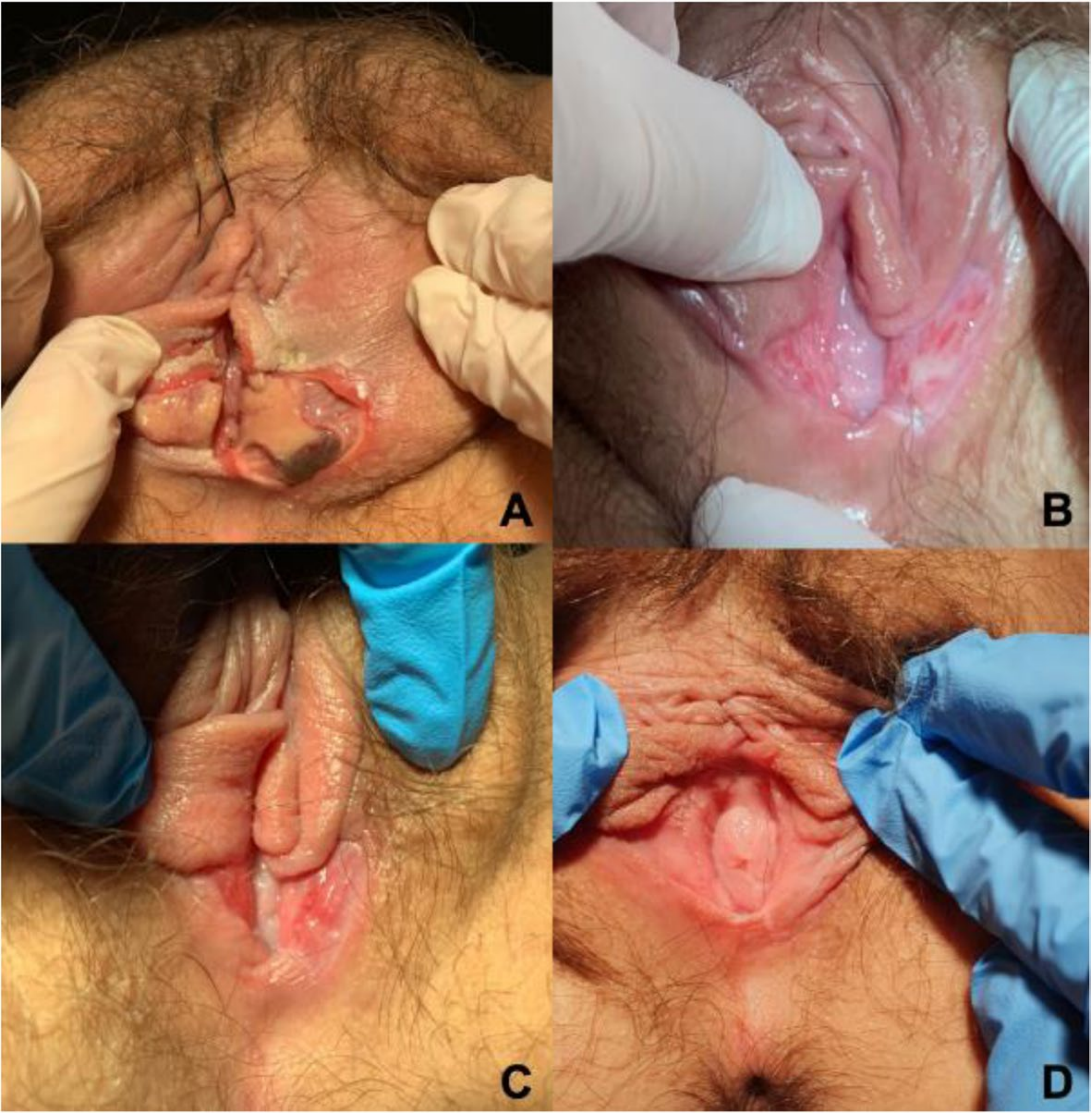
Vulvar ulceration. **A** Day of presentation. **B** Day 16 after presentation. **C** Day 23 after presentation. **D** Day 60 after presentation

The healing process is shown in Fig. 2 Eventually, after 60 days, the lesions completely disappeared, and the appearance of the external genital area returned to normal. The girl remained asymptomatic over a one-year follow-up, during which she was regularly monitored.

The patient had neither previous pathological symptoms of genital area nor a history of sexual intercourse. However, she had a history of autoimmune disorders. She had suffered from recurrent tonsillitis in the course of PFAPA syndrome and IgA vasculitis due to Streptococcus pyogenes bacteriemia. PFAPA manifestations include abdominal pain (similar to that on admission) and swollen cervical lymph nodes (not present on admission).

It is worth mentioning, that she received the first dose of the vaccine against COVID-19 (Comirnaty Pfizer BioNTech (BNT162b2)) a week before the ulceration appeared. At that time, Poland was experiencing the fourth wave of COVID-19.

The patient had not previously suffered from COVID-19. Vulvar pain and fever occurred 2 days after the vaccination. 7 days later, the vulvar ulcers had fully developed. Three months later the patient received the second dose of the vaccine without side effects.

## Discussion

When diagnosing external genital ulcers in women, both endo- and exogenous causes should be considered. The specialists should pay attention to both infectious and non-infectious reasons [9]. Numerous factors such as infectious, inflammatory, immunological, neoplastic, traumatic, or medication-induced causes need to be taken into account [10]. In adolescents the most common causes of AGU are: herpes simplex virus (HSV), Epstein-Barr virus, cytomegalovirus, human immunodeficiency virus (HIV), Behcet’s disease, Crohn’s disease (CD), ulcerative colitis, celiac disease, cyclic neutropenia, periodic fever syndromes and leukemia [9].

### PFAPA syndrome

A thorough physical examination should be performed following detailed history-taking, because a patient’s childhood diseases can have a significant impact on their current health problems. Our patient was diagnosed with PFAPA syndrome in the past. This disease is a complex auto-inflammatory disorder diagnosed on the basis of clinical symptoms such as periodic fever, aphthous stomatitis, pharyngitis, and cervical adenitis. The course of the disease is characterized by alternating periods of relapses and remissions, and the main symptoms are often accompanied by gastrointestinal disorders [11]. Beside the chief complaint, our patient presented abdominal pain, nausea and vomiting.

PFAPA syndrome is associated with a polygenic predisposition to impaired functioning of the innate immune system. The interaction of genetic susceptibility and environmental factors, including infections, predisposes individuals to the occurrence of the disease and its recurrence in the autoimmune mechanism [11, 12]. People suffering from PFAPA display increased activation of CD4Th1 and Th17 lymphocytes [13]. This molecular susceptibility results in the dysfunction of antigen-presenting cells, e.g. monocytes, and in dysregulation of T cells. Monocytes produce higher levels of pro-inflammatory cytokine IL-12, which stimulates CD4 and CD8 lymphocytes to produce excessive amounts of IFN-γ and lipopolysaccharides (LPS) [14]. Moreover, the expression of the IL-10 gene decreases, resulting in a reduction of anti-inflammatory IL-10 cytokine [13]. In addition, down regulation of CCR1 (C–C motif chemokine receptor 1) predisposes to a reduced migration of monocytes [15]. Depletion of these cells in inflammatory diseases may lead to increased migration of microorganisms through the permeable mucous membrane, which results in ulceration [14]. In PFAPA, the classical complement pathway is activated, while the alternative pathway remains unchanged. During PFAPA flare-ups, the levels of T-cell chemokines (IP-10/CXCL10, MIG/CXCL9), G-CSF (granulocyte colony-stimulating factor), and pro-inflammatory cytokines IL-1β, IL-6, IL-12, IL-18 are all increased, and the complement system is activated [11, 12]. PFAPA is a consequence of excessive intracellular protein complexes response with the participation of pro-inflammatory factors such as caspase-1, IL-6, IP-10 (interferon gamma-induced protein 10) and interleukin 1 receptor antagonist (IL1Ra), but the levels of other pro-inflammatory factors, such as TNF **ɑ** (tumor necrosis factor) and MCP-1 (monocyte chemoattractant protein-1), remain relatively steady.

During PFAPA attacks, the complete blood count shows increased levels of neutrophils and monocytes, with low levels of lymphocytes and eosinophils [12, 16]. The disease usually responds to treatment with corticosteroids [11]. The administration of antibiotics is not justified due to the autoimmune basis of the disease. Tonsillectomy provides a 70–97% long-term remission of the PFAPA syndrome [17].

PFAPA syndrome may predispose to the occurrence of AGU [13, 18]. However, AGUs are a very rare and atypical manifestation of PFAPA. The presence of vulvar ulcers in PFAPA patients is influenced by reduced IL-10 (anti-inflammatory cytokine) and CCR1 levels [13, 14]. According to Scattoni et al. this atypical symptom should be regarded as a potential and useful indicator of PFAPA [18].

In the present study, we took into consideration the influence of the PFAPA syndrome on the development of labial ulceration in our patient. The immune and genetic determinants that caused the PFAPA syndrome could also lead to the appearance of ulcers after the occurrence of provoking factors. The patient's leukocyte, neutrophil, and eosinophil levels resembled those typically found in PFAPA syndrome. The vulvar lesions were accompanied by fever episodes, abdominal pain, nausea, and vomiting. Additionally, our patient did not undergo a tonsillectomy, a procedure reducing the risk of the PFAPA syndrome recurrence.

Furthermore, as emphasized by researchers, the incidence of PFAPA syndrome rose during the COVID-19 pandemic [14].

### vaccination against SARS-COV-2

An effective vaccination against SARS-CoV-2 was a vital tool to halt the spread of the pandemic; however, some predisposed people may have presented adverse reactions to the vaccination. Several cutaneous side effects were observed, e.g. delayed large local reactions and eruptions.

Recently, cases of AGU associated with COVID-19 vaccinations have been reported [2, 5, 19]. Of the 94 cases of vulvovaginal ulceration reported in the female adolescent age group, there was evidence that at least 37 were AGUs. In addition, up to December 2022, there were approximately 12 case reports published in scientific literature on genital ulceration after COVID-19 vaccine administration in non-sexually active adolescent patients. In majority, the events occurred after the second dose, usually within 1 week. Common symptoms included pain-related difficulty in urination, defecation, sitting and walking. Fever, vulvar swelling and fatigue were also noted. Despite a different approach, the ulcers were usually self-limiting and healed between 2 to 6 weeks [20].

The most frequently reported altered effects of vaccines are pain and swelling. Mucosal changes (bleeding gums, oral sores and ulcers) may occur after administration of diphtheria, tetanus, acellular pertussis and polio vaccines [19]. Incidences of lichen planus, a chronic inflammatory disease which affects the stratified squamous epithelium and frequently involves the oral and genital mucosa, have been reported after hepatitis B vaccination. In all likelihood, the immune system recognizes epitopes similar/identical to proteins of the virus on keratinocytes and induces immunological response and apoptosis of these cells [21].

Once an mRNA vaccine is administered*,*the spike protein, a viral receptor-binding protein, is produced by ribosomes in muscle cells. Subsequently, it binds to the host receptor angiotensin-converting enzyme 2 and triggers a robust CD8 + and CD4 + cell mediated response, inducing the production of neutralizing antibodies and memory of B and T-cells. COVID-19 vaccinations induce an autoimmune response by several pathways, including the development of specific autoantibodies, the effects of certain vaccine adjuvants, and molecular mimicry [22].

The SARS-CoV-2 spike protein and lung surfactant proteins share 13 out of 24 pentapeptides and the respiratory system is the most frequently attacked system in the case of COVID-19 infection. A similar mechanism of cross-reactions between the virus’ proteins and a variety of human antigens could possibly lead to autoimmunity against other organs, including the formation of mucosal and skin lesions induced by the coronavirus invasion as well as COVID-19 vaccines [22]. The side effects of a vaccination might as well be due to transient bursts of IFN-I expression, effective antibody production, oxidative stress and DNA-damage, which may stimulate hyperinflammatory conditions. Another explanation indicates that in the case of mRNA vaccines, mRNA presents as both antigen and adjuvant, and might be so identified by Toll-like receptors, which trigger inflammation and immunity [22]. Nevertheless, the pathophysiology of AGUs after vaccination remains poorly understood.

A rare problem following COVID-19 vaccination described in the literature is Behcet's disease, and it is also possible that AGUs after vaccination are the first manifestation of this condition [23].

### Behcet’s disease

Behcet’s disease (BD) should also be evaluated in our patient’s history of aphthous lesions in the mouth and genital ulceration. Behcet’s disease is a rare inflammatory disorder diagnosed based on clinical symptoms and specific characteristics. According to the new criteria, a patient who scores ≥ 4 points is classified as having BD. Characteristic signs and symptoms include ocular lesions (2), genital aphthosis (2), oral aphthosis (2), skin lesions (1), neurological manifestations (1), vascular manifestations (1) and, optionally, a positive pathergy test (1) [24]. In addition, BD is often associated with the neutrophil to lymphocyte ratio (NLR) being increased while the hemoglobin (HB) level is decreased. Additionally, there is an increase in the erythrocyte sedimentation rate (ESR) and CRP, and the human leukocyte antigens (HLA-B51) test shows positive results [25].

The pathomechanism of BD may depend on neutrophil-mediated mechanisms, that is, neutrophil hyperactivation via both a massive reactive oxygen species (ROS) production and neutrophil extracellular traps (NETs) release. BD patients have higher serum concentrations of sTNFR, leptin, sCD40L, and IL-6. Tumor necrosis factor alpha (TNF-alpha), leading neutrophils to disrupt the oral mucosa, is elevated in patients with recurrent aphthae, and affects endothelial cell adhesion and neutrophil chemotaxis. This is believed to be one of the molecular factors that are responsible for aphthous ulcers. As mentioned above in the present article, aphthous ulcers may occur both as a rare side effect of COVID-19 vaccination and as a consequence of high serum concentrations of sTNFR in BD [26].

,PFAPA syndrome and Behcet’s disease are characterized by similar pathomechanism. For this reason, they are often considered in the differential diagnosis [10, 13]. It is postulated that the same HLA type, specifically, HLA-B5 and HLA-B51, is involved in the development of both diseases [13, 14]. Thus, ulceration in the vaginal area can be a symptom of PFAPA syndrome and Behcet’s disease.

### Infectious etiology

The vulva can be affected by a variety of microorganisms including bacteria, viruses, fungi and parasites. Commonly, those infections are transmitted by sexual contact. However, in non-sexually active adolescents, genital infections might develop as well. AGU is commonly described as associated with a variety of infections including cytomegalovirus, herpes zoster virus (HZV), influenza type A and B, mumps virus, salmonella, mycoplasma and, most commonly, Epstein-Barr virus [4].

EBV causing infectious mononucleosis syndrome has been reported as a most common cause of AGU. Serologic testing for EBV in patients with vulvar ulcers demonstrated evidence of acute as well as prior infections [3]. Most patients develop systemic symptoms of IMS, and lymphadenopathy distant from the site of ulceration is also common; however, in the presented case the enlargement of lymph nodes has not been noted. Acute CMV infection has also been detected in patients with AGU and CMV inclusions found in cells of the vulva and cervix [3, 27]. As for the vulvar HZV infection, it is uncommon and often causes pain or a burning sensation. Lesions usually appear in a specific dermatomal distribution.

Herpes simplex virus, causing genital herpes, remains the most common factor of genital ulcers among sexually active females. However, it might also be responsible for ulcers in non-sexually active adolescents [4, 28]. There are two types of HSV. HSV-2 is considered to be the main cause of AGU, whilst HSV-1 is mostly linked to oral cavity lesions.

American pediatric and adolescent gynecology care providers suspect an 80% of HSV etiology at the onset of AGU diagnosis, therefore aggressive diagnosis of lesions should be delayed [29].

Since there is no single infectious agent identified as a cause of AGU, clinical examination and detection of viral genetic material or serologic tests play a pivotal role in the diagnostic process.

### Cancer

We also considered the possibility of vulvar cancer, although the incidence of genital cancers are rare in young females [30]. However, the macroscopic appearance of the vulvar lesion as an ulcer raised suspicions of oncological concern. There are two types of vulvar intraepithelial neoplasia (VIN). One of them, defined as differentiated (dVIN), is often associated with lichen sclerosus. The other refers to vulvar high-grade squamous intraepithelial lesions (vH-SIL) [31]. It is an HPV-related oncology condition that is specific to younger women and refers to intraepithelial neoplasia and squamous cell vulvar carcinoma (SVC) [32]. Moreover, given the increase in HPV infections and an early age of sexual initiation, there is a significant risk of vulvar cancer in younger age groups [33]. However, biopsy from vulvar lesions in children is debatable among many researchers unless there is a recurrence or a non-infectious etiology is suspected [29]. Most cases of vulvar ulceration in young women are self-limiting and respond to topical and systemic corticosteroid therapy. For these reasons, some authors recommend reducing extensive diagnostics [34]. In our study, due to the extensive area of necrosis as well as the specificity of a hospital also providing oncological treatment, it was decided to collect material for histopathological examination. Biopsy excluded neoplastic invasion, therefore we ruled out vulvar cancer.

## Conclusion

Genital ulcers are a heterogeneous group, which might be challenging for clinicians due to its possible multidisciplinary background. The cases of rapid appearance and exacerbation, as well as the systemic symptoms of the condition, require efficient diagnostics and treatment. Hence, it is important to know the possible etiology of the disease, as well as to be able to exclude life and health threatening conditions. In the presented case, a rapidly changing condition caused concern, especially regarding its potentially multidisciplinary character. The individual predisposition to autoimmune diseases favored the occurrence of vulvar ulceration and its prolonged convalescence, especially after the action of an additional trigger, namely vaccination against SARS-CoV-2. Our case highlights the importance of past medical history in determining possible correlations between the patient's various clinical conditions, acceleration of the diagnosis and treatment application. Age-specific disease factors should also be examined. In order to fully understand the pathomechanism, etiology and possible prevention in predisposed patients, further research is required.

## Data Availability

All the data for the case reports are available in this manuscript.

## Abbreviations

AGU: Acute genital ulcers/ulceration
SARS-CoV-2: Severe acute respiratory syndrome coronavirus 2
COVID-19: Coronavirus disease of 2019
PFAPA: Periodic fever, aphthous stomatitis, pharyngitis and adenitis
BD: Behcet’s disease
BNT162b2: Comirnaty Pfizer BioNTech
N: Reference range
hpf: Per high power field
WBC: White blood cells
HSV: Herpes simplex virus
VZV: Varicella zoster virus
CMV: Cytomegalovirus
HIV: Human immunodeficiency virus
IL: Interleukin
STAT4: Signal transducer and activator of transcription 4
CCR1-CCR3: C–C motif chemokine receptor 1–3
CD4Th1: Cluster of differentiation 4 protein T helper 1 lymphocytes
Th17: T helper 17 lymphocyte
CD8: Cluster of differentiation 8 protein lymphocyte
IFN-γ: Interferon‐gamma
LPS: Lipopolysaccharides
CASP1: Caspase 1
IL18RAP: Interleukin 18 receptor accessory protein
C1QB: Complement C1q B chain
C2: Complement C2
SERPING1: Serpin family G member 1
AIM2: Interferon-inducible protein absent in melanoma 2
IP-10: Interferon-gamma-induced protein 10
CXCL-10,-9: C-X-C motif chemokine ligand -10,-9
G-CSF: Granulocyte colony-stimulating factor
IL1Ra: Interleukin 1 receptor antagonist
TNFɑ: Tumor necrosis factor ɑ
MCP-1: Monocyte chemoattractant protein-1
mRNA: Messenger ribonucleic acid,
LP: Lichen planus
HLA-B5/B51: Human leukocyte antigen B5/B51
NLR: Neutrophil to lymphocyte ratio
ESR: Erythrocyte sedimentation rate
CRP: C reactive protein
ROS: Reactive oxygen species production
NETs: Neutrophil extracellular traps
sTNFR: Soluble tumor necrosis factor receptor
sCD40L: Soluble form of CD40 ligand
IMS: Infectious mononucleosis syndrome
EBV: Epstein-Barr virus
STI’s: Sexually transmitted infections
VIN: Vulvar intraepithelial neoplasia
dVIN: Differentiated vulvar intraepithelial neoplasia
vH-SIL: Vulvar high-grade squamous intraepithelial lesion
SVC: Squamous cell vulvar carcinoma
